# Enhancing Skin Lesion Detection: A Multistage Multiclass Convolutional Neural Network-Based Framework

**DOI:** 10.3390/bioengineering10121430

**Published:** 2023-12-15

**Authors:** Muhammad Umair Ali, Majdi Khalid, Hanan Alshanbari, Amad Zafar, Seung Won Lee

**Affiliations:** 1 Department of Intelligent Mechatronics Engineering, Sejong University, Seoul 05006, Republic of Korea; umair@sejong.ac.kr; 2 Department of Computer Science and Artificial Intelligence, College of Computers, Umm Al-Qura University, Makkah 21955, Saudi Arabia; mknfiai@uqu.edu.sa (M.K.); hsshanbari@uqu.edu.sa (H.A.); 3 Department of Precision Medicine, Sungkyunkwan University School of Medicine, Suwon 16419, Republic of Korea

**Keywords:** skin lesion detection, skin cancer, convolutional neural network, melanoma, classification

## Abstract

The early identification and treatment of various dermatological conditions depend on the detection of skin lesions. Due to advancements in computer-aided diagnosis and machine learning approaches, learning-based skin lesion analysis methods have attracted much interest recently. Employing the concept of transfer learning, this research proposes a deep convolutional neural network (CNN)-based multistage and multiclass framework to categorize seven types of skin lesions. In the first stage, a CNN model was developed to classify skin lesion images into two classes, namely benign and malignant. In the second stage, the model was then used with the transfer learning concept to further categorize benign lesions into five subcategories (melanocytic nevus, actinic keratosis, benign keratosis, dermatofibroma, and vascular) and malignant lesions into two subcategories (melanoma and basal cell carcinoma). The frozen weights of the CNN developed–trained with correlated images benefited the transfer learning using the same type of images for the subclassification of benign and malignant classes. The proposed multistage and multiclass technique improved the classification accuracy of the online ISIC2018 skin lesion dataset by up to 93.4% for benign and malignant class identification. Furthermore, a high accuracy of 96.2% was achieved for subclassification of both classes. Sensitivity, specificity, precision, and F1-score metrics further validated the effectiveness of the proposed multistage and multiclass framework. Compared to existing CNN models described in the literature, the proposed approach took less time to train and had a higher classification rate.

## 1. Introduction

The skin is the biggest organ in the human body, which also functions as a barrier against heat, light, and infections. In addition to protecting the body, it is essential for controlling body temperature and storing fat and water [1]. The epidermis, dermis, and subcutaneous fat are the three primary layers [2]. Skin cancer begins in the cells, which are the essential building components of the skin. Skin cells grow and divide naturally, replacing old cells with new ones as part of the body’s normal process. This natural cycle occasionally breaks down. When the skin does not require new cells, they form, and existing cells die when they should not. These extra cells build up and form a tissue mass known as a tumor [3,4]. 

Skin lesions are commonly classified into two classes: malignant (melanoma (MEL) and basal cell carcinoma (BCC)) and benign (melanocytic nevus (NV), actinic keratosis (AK), benign keratosis (BKL), dermatofibroma (DF), and vascular (VASC)) [5,6]. The majority of skin cancer-related deaths are caused by MEL and BCC, which are the most aggressive and deadly types of the disease. The specific cause remains mysterious despite continuous investigation [4,7]. However, this condition develops due to various elements, including environmental factors, UV radiation exposure, and genetic predisposition. According to Seigel [8], the estimated new skin cancer cases in the United States are around 104,930 (62,810 are male and 42,120 are female), with around 12,470 deaths (8480 are male and 3990 are female).

Even though malignant skin cancer has a very high survival rate when diagnosed early, its widespread prevalence remains a major societal concern. Melanoma can spread through the lymphatic or circulatory systems, reaching distant parts of the body in some situations. Among the numerous skin cancer forms, this cancer has the highest risk of spreading [9,10]. According to research, early identification considerably reduces melanoma-related mortality rates [11]. Even for specialists, early diagnosis remains challenging. Simplifying the diagnosis process using novel technologies could benefit healthcare workers.

A non-invasive imaging method called dermoscopy has been developed to diagnose skin cancer more accurately during clinical examinations [12]. The dermoscopy devices can help differentiate between benign and malignant skin lesions because of their high visual perception. Dermatologists are now better able to distinguish between malignant and benign images because of the development of numerous conventional methods, such as the Menzies technique [13], the ABCD rule [14], the seven-point checklist [15], and CASH [16]. Accurate diagnosis of skin cancer by an expert is difficult due to intra-class similarities. Furthermore, the color, size, and other features of skin cancer types are very similar. Image processing and machine vision use for various medical imaging applications has grown tremendously in the past decade [17,18,19,20,21,22]. Using these strategies speeds up the diagnosis process and reduces human error. Utilizing the proven effectiveness of machine learning and deep learning techniques in various applications [23,24], the researchers used these techniques on dermoscopy images to examine skin lesions [25,26]. Since 2015, dermoscopic image analysis (DIA) has relied primarily on convolutional neural networks (CNNs) as classifiers, with advanced computer-aided diagnosis research emphasizing the importance of CNN in achieving superior results in image classification, detection, and segmentation in complex scenarios [27]. Codella et al. [26] investigated popular deep neural network models, such as deep residual and CNN models, to identify malignant lesions. Thomas et al. [28] classified tissues into 12 dermatologist classes using a CNN framework for skin lesion detection. They outperformed clinical accuracy by achieving a high accuracy of 97.9% compared to 93.6% for the clinical technique. Amin et al. [29] designed a framework to compute deep features. They employed methods such as image scaling, biorthogonal 2D wavelet transform, the Otsu algorithm, RGB-to-luminance channel conversion, and pretrained networks such as VGG16 and AlexNet. Principal component analysis was applied to choose the best features for categorization. Al-Masni et al. [30] designed a full-resolution convolutional network for the segmentation of dermoscopic images. The results showed that the ResNet-50 pretrained model had the best accuracy compared to others. Another study found that the SENet CNN can be used to detect skin lesions, and its proposed model had a high detection rate of 91% for the ISIC2019 dataset [31]. Recently, Bibi et al. [32] proposed a deep feature fusion-based framework to categorize dermoscopic images into subclasses. They used DensNet-201 and DarkNet-53 CNNs to extract the deep features after applying the contrast enhancement approach. A genetic optimization algorithm was used to select the optimal parameters for learning of the models, and the serial–harmonic mean approach was used to fuse the features of both models. The marine predator-based optimization algorithm was employed to discard the irrelevant features. They used ISIC2018 (https://challenge.isic-archive.com/data/#2018) and ISIC2019 (https://challenge.isic-archive.com/data/#2019) online datasets to validate their proposed framework and achieved a high classification accuracy of 85.4% and 98.80%, respectively. Although their models showed high performance, the computational time was also increased due to pretrained models’ training and irrelevant feature removal. Therefore, further research is still needed to achieve high performance with low training time and categorize the subclasses of skin lesions with a high classification rate to assist doctors in making early treatment decisions. The following are the main contribution of this study:

- A new multistage and multiclass identification CNN-based framework for skin lesion detection using dermoscopic images is presented;
- First, an isolated CNN was developed from scratch to classify the dermoscopic images into malignant and benign classes;
- Second, the developed isolated CNN model was used to develop two new CNN models to further classify each detected class (malignant and benign) into subcategories (MEL and BCC in the case of malignant and NV, AK, BK, DF, and VASC in the case of benign) using the idea of transfer learning. It was hypothesized that the frozen weights of the CNN developed and trained on correlated images could enhance the effectiveness of transfer learning when applied to the same type of images for subclassifying benign and malignant classes;
- The online skin lesions dataset was used to validate the proposed framework;
- The proposed multistage and multiclass framework results were also compared with the existing pretrained models and the literature.

## 2. Proposed Framework

**Figure 1:**
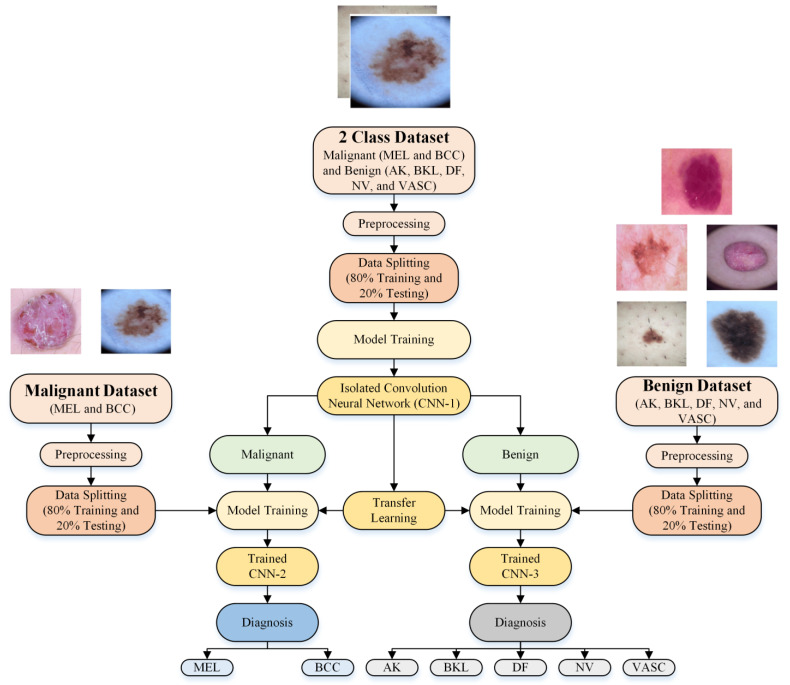
A proposed deep learning network-based multistage and multiclass framework for skin lesion detection.

Figure 1 depicts the proposed multistage and multiclass framework for skin lesion detection using isolated and deep transfer learning models. The dermoscopic images were preprocessed to minimize noise and adjust the size. The isolated CNN model (CNN-1) was then developed to classify the dermoscopic images into two categories (benign and malignant). Two new deep learning models (CNN-2 and CNN-3) were built from the CNN-1 using transfer learning to further categorize each class type into subclasses (MEL and BCC in the case of malignant (CNN-2 model) and NV, AK, BK, DF, and VASC in the case of benign (CNN-3 model)). The frozen weights of the trained CNN-1 from correlated images benefited the transfer learning for the same type of images for the subclassification of benign and malignant classes. The subsequent sections provide a detailed explanation of each step.

### 2.1. Dataset Description

This work used an online skin lesions dataset to validate the proposed CNN-based multistage and multiclass framework [5]. The dataset used for skin cancer classification is HAM10000 and publically available (https://challenge.isic-archive.com/data/#2018, accessed on 1 November 2023); it consists of dermatoscopic images of a diverse range of skin lesions. The dataset includes 10,015 high-resolution dermatoscopic images collected over two decades from two separate locations: the Department of Dermatology at the Medical University of Vienna, Austria, and Cliff Rosendahl’s skin cancer practice in Queensland, Australia [5]. Professional dermatologists have annotated clinical diagnoses to the dataset, offering trustworthy reference data for machine learning model training and assessment. However, challenges such as imbalanced class distribution, noise, and the existence of undesired areas pose obstacles to developing models with robust generalization across all lesion types. Further details about the samples in various classes are presented in Table 1. 

The MEL and BCC classes belong to the malignant category, and the remaining belong to the benign category (NV, AK, BK, DF, and VASC). Further details about the dataset can be found in [5].

### 2.2. Preprocessing

Extraneous information is included in dermoscopic images, following a low categorization rate. To improve relevance, it is critical to remove noise and undesirable regions. The cropping approach is used to estimate extreme points, while noise-reduction techniques such as erosion and dilatation are used to reduce undesirable elements [19,33]. The data augmentation was also applied to adjust the size (to 227 × 227) and balance the dataset (1000 samples per class) using rotation and translation. 

### 2.3. Development of CNN Models

An isolated CNN is meant to train for a specific task without prior knowledge [34]. A transfer-learned model, on the other hand, uses knowledge from pre-existing models [35]. Transfer learning entails training a base model for subsequent tasks utilizing base images. The new CNN is then trained by combining previously learned features from a previously trained CNN that has been precisely tuned for the new task [36]. Pretrained and newly designed CNNs are the two most common techniques for transfer learning [22,37]. Publicly accessible pretrained models like ResNet50, ShuffleNet, SqueezeNet, MobileNet v2, and GoogleNet can be modified for a particular task. On the other hand, newly developed networks are built from scratch, reutilizing neuron weights by modifying particular CNN model layers to fit the objective task.

The isolated CNN was designed to classify dermoscopic images into malignant and benign categories. After that, the developed isolated CNN model was reused to subcategorize both classes. 

#### 2.3.1. Isolated CNN for Binary Class Classification

A CNN is made up of multiple layers, including an input layer and a processing layer, including convolutional, ReLU, and pooling layers. These layers work together to retrieve various pieces of information from an image. A fully connected layer then uses the collected features to classify the image [36,38]. A CNN also includes neurons, weights, bias factors, and activation functions in addition to layers.

**Figure 2:**
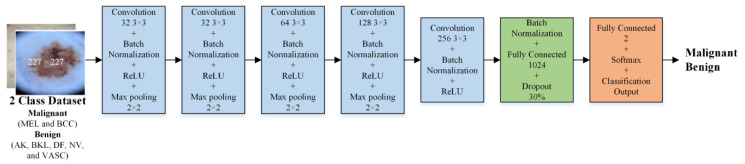
The isolated CNN (CNN-1) developed to categorize the skin dermoscopic images into two classes (malignant and benign).

In this research, an isolated CNN was designed to categorize dermoscopic images of skin into binary classes (malignant and benign). Different isolated CNN models were developed to evaluate their performance. The isolated CNN model’s input layer comprised pixel values taken from images. Notably, the 26-layer isolated CNN model (CNN-1) outperformed the others in binary classification. As a result, Figure 2 depicts the detailed architecture of this high-performing model and the relevant parameters.

#### 2.3.2. Developed Transfer Learned CNNs for Subcategorization

**Figure 3:**
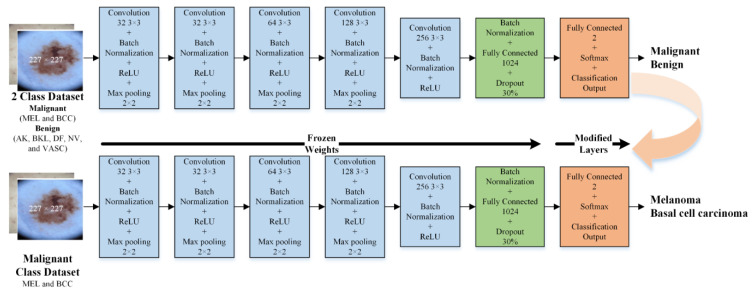
The CNN (CNN-2) developed using transfer learning to categorize the dermoscopic images into two malignant classes (MEL and BCC).

**Figure 4:**
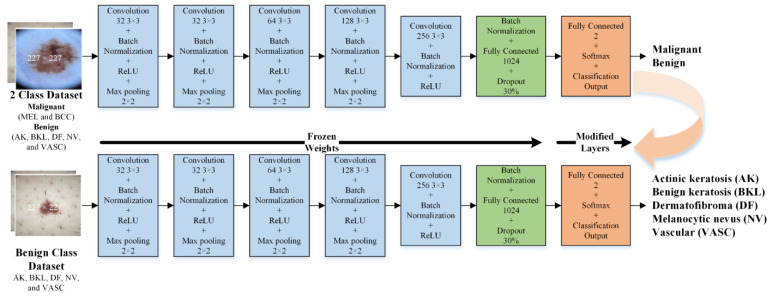
The isolated CNN (CNN-3) developed to categorize the dermoscopic images into five benign classes (AK, BKL, DF, NV, and VASC).

This research applied transfer learning using a developed CNN, as explained in the previous sections. Reusing the CNN-1 model developed for binary classes (malignant and benign), two different CNN models were retrained by exchanging the final three layers, as shown in Figure 3 and Figure 4. CNN-2 was developed to further classify the malignant class into MEL and BCC. Figure 3 shows the detailed architecture of CNN-2. Similarly, one more CNN model (CNN-3) was developed to subclassify the benign class into AK, BKL, DF, NF, and VASC. Figure 4 shows the detailed architecture of CNN-3.

#### 2.3.3. CNN Optimization

By lowering the cost/loss function, optimization plays a critical part in improving the accuracy of CNNs. Optimization measures the extent to which learnable parameters have been computed, and loss reduction has been achieved.

**Figure 5:**
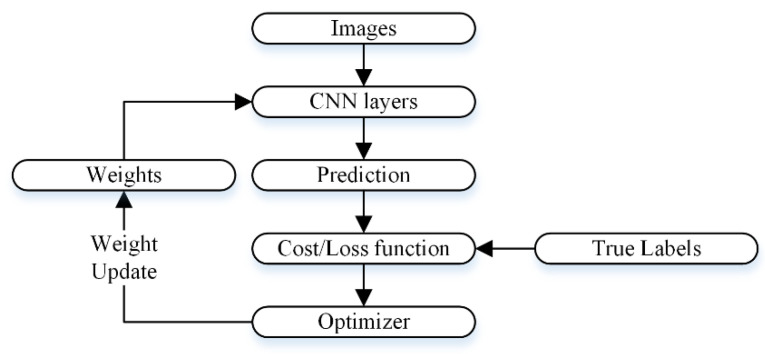
The workflow for updating the CNN’s weights.

To compute image features, convolution layer filters employ parameters that are learned. During training, these parameters are initialized randomly. Each epoch’s loss is determined by the target and predicted class labels. In the subsequent epoch, the optimizer updates the learnable parameters, constantly updating them to minimize the loss. Figure 5 depicts the working of the optimizer. The stochastic gradient descent with momentum (SGDM) method was used for optimization in this work.

## 3. Results

In this study, all simulations and analyses were conducted using MATLAB 2023a on a personal computer with the following specifications: core i7, 12th generation, 32 GB RAM, NVIDIA GeForce RTX 3050, 1 TB SSD, and a 64-bit Windows 11 operating system. For each CNN training, the following parameters were selected: 100 epochs, 0.9 momentum, 128 mini batch-size, and 0.001 learning rate.

First, the augmentation was performed to balance the ISIC2018 skin lesion dataset. After performing the augmentation, each of the seven classes had 1000 samples per class. The dataset was split into 80:20 ratios for CNN training and testing. The images used for model testing were not used to train the CNN. Various commonly publically available pretrained CNNs, such as ResNet50, Inception V3, GoogleNet, and DenseNet-201, were used to categorize the skin lesions dataset. The results of all mentioned pretrained models and developed 26-layer CNN are presented in Table 2. 

**Figure 6:**
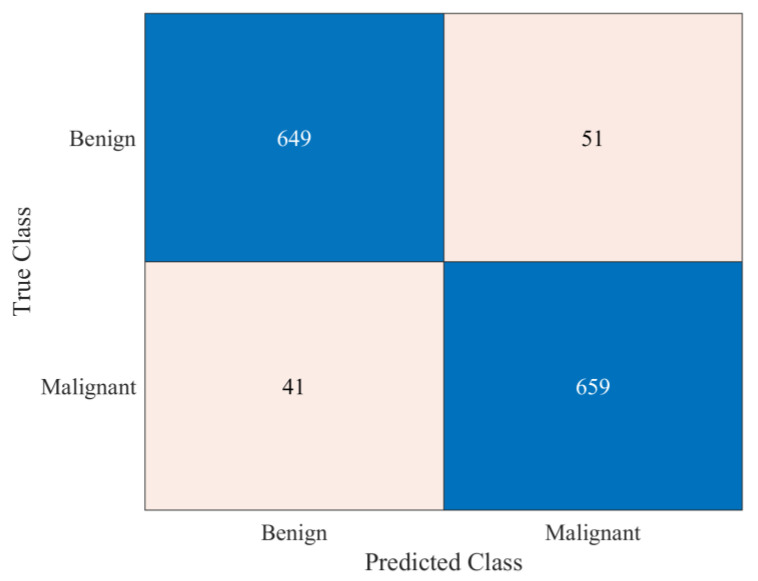
Performance of the CNN-1 developed for binary classification.

After analyzing the results presented in Table 2, it was evident that all the pretrained models showed a reasonable classification performance, but the time taken for training was relatively high. The developed 26-layer CNN model took less training time but produced a low classification rate compared to pretrained models. Therefore, this work used a multistage and multiclass framework for skin lesion detection using isolated and deep transfer learning models. First, all the classes were grouped into two classes, namely benign and malignant. The benign class had all the images of AK, BKL, DF, NV, and VASC, whereas the malignant group contained the images of MEL and BCC classes. CNN-1 was trained to classify the dermoscopic images into binary classes. The performance of the CNN-1 model is illustrated in Table 3 and Figure 6. 

**Figure 7:**
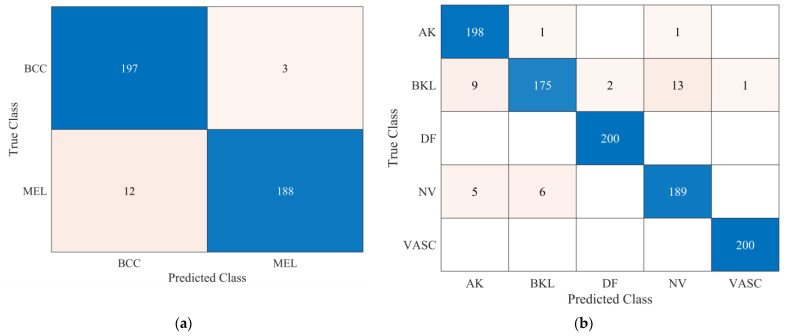
(**a**) Performance of the CNN-2 model for subclassification of malignant class; (**b**) performance of the CNN-2 model for subclassification of benign class.

It is evident from the results presented in Table 3 and Figure 6 that the developed CNN-1 model detected the benign and malignant classes with a high accuracy of 93.4% using dermoscopic images. It correctly classified the 649 images out of 700 for the benign class and had a high true positive rate of 92.7%. Similarly, the 659 images of the malignant class were correctly classified using the developed CNN-1 model. It also showed a high classification rate of 94.1%, with a low false negative rate of only 5.9%. To further classify each class into subclasses, the CNN-2 and CNN-3 models were developed for malignant and benign classes using the idea of transfer learning, respectively, as discussed above. The results of both developed CNN transfer learned models are presented in Table 4 and Figure 7.

**Figure 8:**
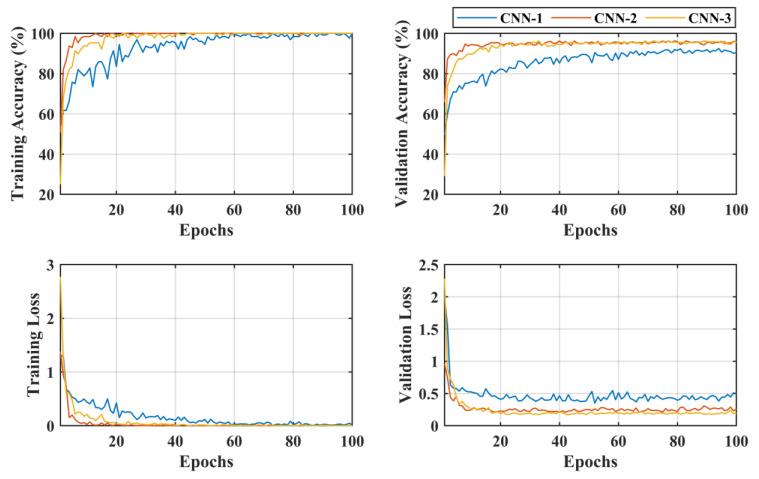
Learning curves of the proposed multistage multiclass framework.

The CNN-2 classifies the malignant class with a high accuracy of 96.25%, with a true positive rate (sensitivity) of 98.5% and 94% for the BCC and MEL classes, respectively. Similarly, in the case of benign class subclassification, the CNN-3 showed a high accuracy performance of 96.2% for five class classification problems. The VASC class was correctly classified with 100% accuracy, whereas the DF class also showed the same classification rate of 100% accuracy. BKL class had the lowest true positive rate (sensitivity) of 87.5% only, with a 12.5% false negative rate. The positive predictive values (precision) were 93.4%, 96.2%, 99%, 93.1%, and 99.5% for the AK, BKL, DF, NV, and VASC, respectively. The learning curves of the proposed multistage multiclass framework are presented in Figure 8. After carefully analyzing the learning curves, it was found that the CNN-1 was stable for almost 60 epochs. In contrast, the CNN-2 and CNN-3 reached 100% training and validation accuracy after 20 epochs. This validated the proposed multistage multiclass framework’s robustness and high classification performance. 

**Figure 9:**
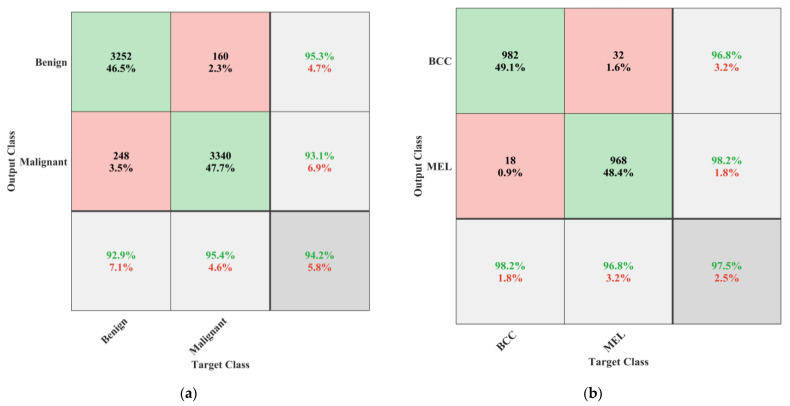

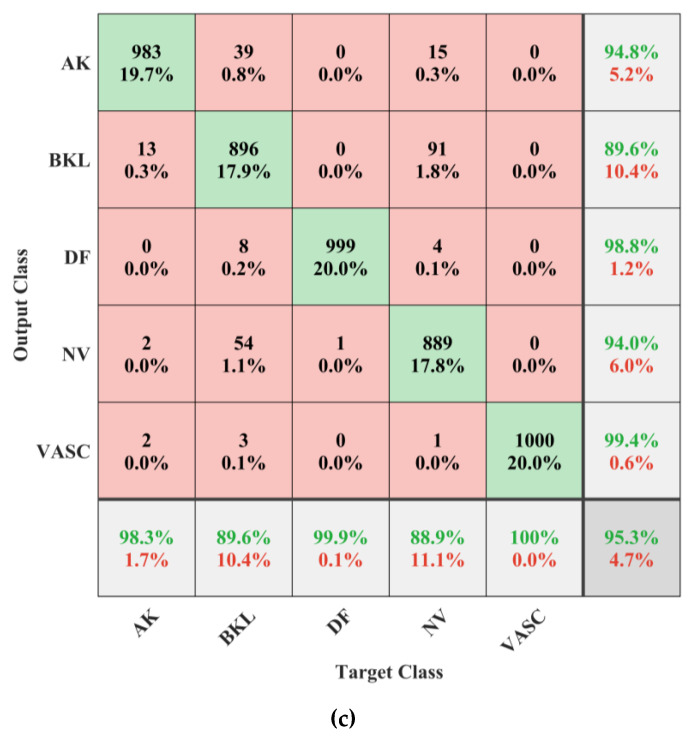
Performance of the proposed multistage multiclass approach: (**a**) CNN-1; (**b**) CNN-2; (**c**) CNN-3.

To further validate the performance of the proposed multistage multiclass approach, the results of 10-fold cross-validation are shown in Figure 9.

## 4. Discussion

Skin cancer, a common and potentially fatal condition, is typically classified as benign or malignant. Benign lesions are often low-risk; however, malignant lesions, such as MEL and BCC, can be fatal. 

This research focuses on improving these classifications by employing multistage and multiclass CNN-based framework to attain noteworthy accuracy in subclassifying malignant and benign skin lesions. In the first stage, the classes were classified as benign or malignant. The developed CNN-1 model achieved a high binary classification accuracy of 93.4%, excelling in detecting benign and malignant classes with minimal false negative rates (see Table 3 and Figure 6). The ablation study was carried out before finalizing the layers of developed CNN—the results of the ablation study are presented in Table 5. 

The ablation study findings show the effect of changing the number of layers in the developed CNNs. It shows that as the number of layers extends from 22 to 34, the training loss reduces, with the 30-layer CNN having the lowest value. Meanwhile, training accuracy stays steady (100%), implying that deeper networks may match the training data more closely, resulting in superior training performance. With an increase in layers, the trend in validation loss does not decrease. Validation losses are lower for the 26-layer and 30-layer CNNs than for the 22-layer and 34-layer models. The 26-layer and 30-layer models seem to provide greater generalization to unknown data, which is reflected in increased validation accuracy. As expected, the training time rises with the number of layers. Deeper networks can take longer to train due to increased computational complexity. The 26-layer CNN surpasses the 22-layer, 30-layer, and 34-layer models in terms of validation accuracy (93.4%). It implies that an ideal balance of model complexity and generalization is obtained with 26 layers since too few or too many layers may result in suboptimal validation data performance. Therefore, the 26-layer CNN model was selected. 

CNN-2 and CNN-3 models were introduced using a newly developed 26-layer CNN built from scratch (CNN-1), reutilizing neuron weights by modifying particular layers for additional subclassification, with outstanding accuracy rates of 96.2% for both malignant and benign subclasses (see Table 4). Figure 7a,b depict CNN-2 and CNN-3 performance in subclassifying malignant and benign classes, respectively. CNN-2 achieved 96.2% accuracy, with noteworthy sensitivity for BCC and MEL classes. CNN-3 subclassified benign lesions with 96.2% accuracy, and high precision across all classes. The comparison of the proposed approach with the latest literature is presented in Table 6. 

In Table 6, it can be seen that the proposed framework yielded the best classification performance compared to others. Budhiman et al. [39] used the ResNet 50 pretrained model to classify the skin images into two classes, and it had a correct classification rate of only 87%. In [40], the multiscale multi-CNN approach was used for skin lesion detection and reported an accuracy of 86.2%. Their model yielded a reasonable accuracy and had a high training time. In another study [45], the authors extracted the local and global level features and fused them with deep features to detect melanoma. The model showed high classification accuracy. However, it could only classify the dermoscopic images into normal and melanoma classes. In addition, the authors did not consider any feature selection method to remove the redundant features. In contrast, in [32], the deep features were extracted using the DensNet-201 and DarkNet-53, and a marine predator optimizer was applied to extract the useful features, an approach that yielded an accuracy of 85.4% for seven class ISIC2018 datasets. Furthermore, Mehwish et al. [44] used a wrapper-based approach to remove the redundant deep features and reported a high accuracy of 92.01%. However, the feature selection approach with CNN can enhance the complexity and compatibility issues with dependencies on the external algorithms. Therefore, this study proposes a multistage and multiclass CNN-based framework; it shows a high classification rate with minimal training time compared to pretrained CNNs (see Table 2, Table 3 and Table 4), paving the way for improved skin lesion identification and subcategorization.

In this study, the CNN hyperparameter was not fine-tuned, and augmentation was applied to balance the datasets. However, in the future, the fine-tuning of the CNN hyperparameter and original dermoscopic images may be considered to evaluate the proposed framework’s performance further. In addition, this study utilized a simple architecture; however, more intuitive architectures like natural language processing may be tested in the future.

## 5. Conclusions

The present study proposed a new multistage and multiclass CNN-based framework for skin lesion detection using dermoscopic images. First, a 26-layer CNN (CNN-1) was developed from scratch to distinguish between benign and malignant images, and the CNN-1 achieved a high classification rate of 93.4% and only took 11 min and 41 s for model training. After that, two new CNN models (CNN-2 and CNN-3) were developed for the subclassification of each identified class. Both models were developed by reutilizing the weights of CNN-1 using transfer learning. Both models showed promising classification accuracy for subcategorizing benign and malignant classes with a very low training time. Both the trained models showed a high classification rate of 96.2% for BCC and MEL (in the case of CNN-2) and AK, BKL, DF, NV, and VASC (in the case of CNN-3) classes. The results were also compared in terms of accuracy and training time with those of various pretrained models. The final results demonstrated that employing the proposed multistage multiclass CNN-based framework yielded the best skin lesion detection.

## Figures and Tables

**Table 1:** Details of ISIC2018 skin lesions dataset.

Types	Dermoscopic Images	No. of Samples
MEL	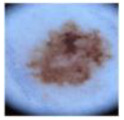	1113
BCC	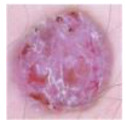	514
AK	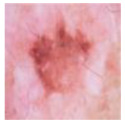	327
BKL	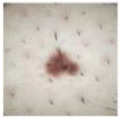	1099
DF	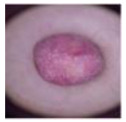	115
NV	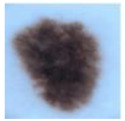	6705
VASC	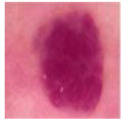	142

**Table 2:** Performance of pretrained CNNs for ISIC2018 dataset.

Parameters		CNNs				
		ResNet50	Inception V3	GoogleNet	DenseNet-201	26-Layer CNN
Training Loss		0.0019	0.0011	0.0011	0.0012	0.0016
Training Accuracy (%)		100	100	100	100	100
Validation Loss		0.3425	0.4971	0.4971	0.3526	0.5216
Validation Accuracy (%)		92.42	91.57	91.57	93.1	90.07
Training Time		389 min 46 s	513 min 43 s	67 min 11 s	1227 min 29 s	11 min 43 s
Sensitivity	AK	0.98	0.96	0.96	0.985	0.985
	BCC	0.945	0.935	0.935	0.935	0.920
	BKL	0.885	0.82	0.82	0.905	0.795
	DF	1	1	1	1	1
	MEL	0.76	0.865	0.865	0.855	0.825
	NV	0.9	0.83	0.83	0.84	0.780
	VASC	1	1	1	1	1
Specificity	AK	0.987	0.991	0.991	0.993	0.980
	BCC	0.995	0.985	0.985	0.993	0.992
	BKL	0.977	0.988	0.988	0.978	0.977
	DF	0.998	0.995	0.995	0.998	0.998
	MEL	0.981	0.967	0.967	0.980	0.964
	NV	0.975	0.978	0.978	0.978	0.978
	VASC	1	0.998	0.998	0.999	0.996
Precision	AK	0.925	0.946	0.946	0.961	0.891
	BCC	0.969	0.912	0.912	0.959	0.948
	BKL	0.863	0.916	0.916	0.874	0.850
	DF	0.985	0.971	0.971	0.985	0.990
	MEL	0.869	0.812	0.812	0.877	0.793
	NV	0.857	0.865	0.865	0.866	0.852
	VASC	1	0.99	0.99	0.995	0.976
F1 Score	AK	0.952	0.953	0.953	0.973	0.936
	BCC	0.957	0.923	0.923	0.947	0.934
	BKL	0.874	0.865	0.865	0.889	0.822
	DF	0.992	0.985	0.985	0.993	0.995
	MEL	0.811	0.838	0.838	0.866	0.809
	NV	0.878	0.847	0.847	0.853	0.815
	VASC	1	0.995	0.995	0.998	0.988

**Table 3:** Performance of developed CNN-1 model for binary classification.

Parameters		CNN-1
Training Loss		0.0074
Training Accuracy (%)		100
Validation Loss		0.4563
Validation Accuracy (%)		93.4
Training Time		11 min 41 s
Sensitivity	Benign	0.927
	Malignant	0.941
Specificity	Benign	0.941
	Malignant	0.927
Precision	Benign	0.941
	Malignant	0.928
F1 Score	Benign	0.934
	Malignant	0.935

**Table 4:** Performance of developed CNN-2 and CNN-3 models.

Parameters		CNNs	
		CNN-2	CNN-3
Training Loss		3.73 × 10^−4^	2.63 × 10^−3^
Training Accuracy (%)		100	100
Validation Loss		0.2576	0.1956
Validation Accuracy (%)		96.25	96.20
Training Time		389 min 46 s	513 min 43 s
Sensitivity	AK	-	0.990
	BCC	0.985	-
	BKL	-	0.875
	DF	-	1
	MEL	0.940	-
	NV	-	0.945
	VASC	-	1
Specificity	AK	-	0.983
	BCC	0.940	-
	BKL	-	0.991
	DF	-	0.998
	MEL	0.985	-
	NV	-	0.983
	VASC	-	0.999
Precision	AK	-	0.934
	BCC	0.943	-
	BKL		0.962
	DF	-	0.990
	MEL	0.984	-
	NV	-	0.931
	VASC	-	0.995
F1 Score	AK	-	0.961
	BCC	0.963	-
	BKL	-	0.916
	DF	-	0.995
	MEL	0.962	-
	NV	-	0.938
	VASC	-	0.998

**Table 5:** Results of ablation study.

Parameters	Developed CNNs			
	22-Layer	26-Layer	30-Layer	34-Layer
Training Loss	0.0412	0.0074	0.0051	0.0264
Training Accuracy (%)	98.43	100	100	100
Validation Loss	0.5645	0.4563	0.498	0.5549
Validation Accuracy (%)	89.7	93.4	92.2	90.5
Training Time	11 min 10 s	11 min 41 s	13 min 35 s	13 min 43 s

**Table 6:** Comparison of the proposed multistage and multiclass CNN with the literature.

Study	Accuracy (%)
Budhiman et al. [39]	87 (for normal and melanoma class)
Bibi et al. [32]	85.4
Mahbod et al. [40]	86.2
Ali et al. [41]	87.9
Carcagnì et al. [42]	88
Sevli [43]	91.51
Mehwish et al. [44]	92.01
Bansal et al. [45]	94.9 (for normal and melanoma class)
This study	93.4 (for benign and malignant) 94.2 (for benign and malignant using 10-fold cross-validation) 96.2 (for subclassification of benign and malignant) 97.5 (for subclassification of malignant using 10-fold cross-validation) 95.3 (for subclassification of benign using 10-fold cross-validation)

## Data Availability

The datasets used in this work are publically available (https://challenge.isic-archive.com/data/#2018, accessed on 1 November 2023).

## References

[1] Byrd A.L., Belkaid Y., Segre J.A. (2018). The human skin microbiome. Nat. Rev. Microbiol..

[2] Gordon R. (2013). Skin Cancer: An Overview of Epidemiology and Risk Factors. Semin. Oncol. Nurs..

[3] O’Sullivan D.E., Brenner D.R., Demers P.A., Villeneuve P.J., Friedenreich C.M., King W.D. (2019). Indoor tanning and skin cancer in Canada: A meta-analysis and attributable burden estimation. Cancer Epidemiol..

[4] Zhang N., Cai Y.-X., Wang Y.-Y., Tian Y.-T., Wang X.-L., Badami B. (2020). Skin cancer diagnosis based on optimized convolutional neural network. Artif. Intell. Med..

[5] Tschandl P., Rosendahl C., Kittler H. (2018). The HAM10000 dataset, a large collection of multi-source dermatoscopic images of common pigmented skin lesions. Sci. Data.

[6] Griffiths C.E., Barker J., Bleiker T.O., Chalmers R., Creamer D. (2016). Rook's Textbook of Dermatology, 4 Volume Set.

[7] Yaiza J.M., Gloria R.A., Belén G.O.M., Elena L.-R., Gema J., Antonio M.J., Ángel G.C.M., Houria B. (2019). Melanoma cancer stem-like cells: Optimization method for culture, enrichment and maintenance. Tissue Cell.

[8] Siegel R.L., Miller K.D., Wagle N.S., Jemal A. (2023). Cancer statistics, 2023. CA Cancer J. Clin..

[9] Dalila F., Zohra A., Reda K., Hocine C. (2017). Segmentation and classification of melanoma and benign skin lesions. Optik.

[10] Razmjooy N., Sheykhahmad F.R., Ghadimi N. (2018). A hybrid neural network—World cup optimization algorithm for melanoma detection. Open Med..

[11] Silveira M., Nascimento J.C., Marques J.S., Marcal A.R.S., Mendonca T., Yamauchi S., Maeda J., Rozeira J. (2009). Comparison of Segmentation Methods for Melanoma Diagnosis in Dermoscopy Images. IEEE J. Sel. Top. Signal Process..

[12] Fargnoli M.C., Kostaki D., Piccioni A., Micantonio T., Peris K. (2012). Dermoscopy in the diagnosis and management of non-melanoma skin cancers. Eur. J. Dermatol..

[13] Argenziano G., Soyer H.P., Chimenti S., Talamini R., Corona R., Sera F., Binder M., Cerroni L., De Rosa G., Ferrara G. (2003). Dermoscopy of pigmented skin lesions: Results of a consensus meeting via the Internet. J. Am. Acad. Dermatol..

[14] Nachbar F., Stolz W., Merkle T., Cognetta A.B., Vogt T., Landthaler M., Bilek P., Braun-Falco O., Plewig G. (1994). The ABCD rule of dermatoscopy: High prospective value in the diagnosis of doubtful melanocytic skin lesions. J. Am. Acad. Dermatol..

[15] Kawahara J., Daneshvar S., Argenziano G., Hamarneh G. (2019). Seven-Point Checklist and Skin Lesion Classification Using Multitask Multimodal Neural Nets. IEEE J. Biomed. Health Inform..

[16] Henning J.S., Dusza S.W., Wang S.Q., Marghoob A.A., Rabinovitz H.S., Polsky D., Kopf A.W. (2007). The CASH (color, architecture, symmetry, and homogeneity) algorithm for dermoscopy. J. Am. Acad. Dermatol..

[17] Shoaib Z., Akbar A., Kim E.S., Kamran M.A., Kim J.H., Jeong M.Y. (2023). Utilizing EEG and fNIRS for the detection of sleep-deprivation-induced fatigue and its inhibition using colored light stimulation. Sci. Rep..

[18] Shoaib Z., Chang W.K., Lee J., Lee S.H., Phillips V.Z., Lee S.H., Paik N.-J., Hwang H.-J., Kim W.-S. (2023). Investigation of neuromodulatory effect of anodal cerebellar transcranial direct current stimulation on the primary motor cortex using functional near-infrared spectroscopy. CerebellumPl.

[19] Ali M.U., Kallu K.D., Masood H., Tahir U., Gopi C.V.V.M., Zafar A., Lee S.W. (2023). A CNN-Based Chest Infection Diagnostic Model: A Multistage Multiclass Isolated and Developed Transfer Learning Framework. Int. J. Intell. Syst..

[20] Ali M.U., Hussain S.J., Zafar A., Bhutta M.R., Lee S.W. (2023). WBM-DLNets: Wrapper-Based Metaheuristic Deep Learning Networks Feature Optimization for Enhancing Brain Tumor Detection. Bioengineering.

[21] Zafar A., Hussain S.J., Ali M.U., Lee S.W. (2023). Metaheuristic Optimization-Based Feature Selection for Imagery and Arithmetic Tasks: An fNIRS Study. Sensors.

[22] Alanazi M.F., Ali M.U., Hussain S.J., Zafar A., Mohatram M., Irfan M., AlRuwaili R., Alruwaili M., Ali N.H., Albarrak A.M. (2022). Brain Tumor/Mass Classification Framework Using Magnetic-Resonance-Imaging-Based Isolated and Developed Transfer Deep-Learning Model. Sensors.

[23] Huang X.-L., Ma X., Hu F. (2018). Editorial: Machine Learning and Intelligent Communications. Mob. Netw. Appl..

[24] Cerquitelli T., Meo M., Curado M., Skorin-Kapov L., Tsiropoulou E.E. (2023). Machine learning empowered computer networks. Comput. Netw..

[25] Zafar M., Sharif M.I., Sharif M.I., Kadry S., Bukhari S.A.C., Rauf H.T. (2023). Skin Lesion Analysis and Cancer Detection Based on Machine/Deep Learning Techniques: A Comprehensive Survey. Life.

[26] Debelee T.G. (2023). Skin Lesion Classification and Detection Using Machine Learning Techniques: A Systematic Review. Diagnostics.

[27] Zeiler M.D., Fergus R. Visualizing and understanding convolutional networks. Proceedings of the Computer Vision—ECCV 2014: 13th European Conference.

[28] Thomas S.M., Lefevre J.G., Baxter G., Hamilton N.A. (2021). Interpretable deep learning systems for multi-class segmentation and classification of non-melanoma skin cancer. Med. Image Anal..

[29] Amin J., Sharif A., Gul N., Anjum M.A., Nisar M.W., Azam F., Bukhari S.A.C. (2020). Integrated design of deep features fusion for localization and classification of skin cancer. Pattern Recognit. Lett..

[30] Al-masni M.A., Kim D.-H., Kim T.-S. (2020). Multiple skin lesions diagnostics via integrated deep convolutional networks for segmentation and classification. Comput. Methods Programs Biomed..

[31] Pacheco A.G., Ali A.-R., Trappenberg T. (2019). Skin cancer detection based on deep learning and entropy to detect outlier samples. arXiv.

[32] Bibi S., Khan M.A., Shah J.H., Damaševičius R., Alasiry A., Marzougui M., Alhaisoni M., Masood A. (2023). MSRNet: Multiclass Skin Lesion Recognition Using Additional Residual Block Based Fine-Tuned Deep Models Information Fusion and Best Feature Selection. Diagnostics.

[33] Rosebrock A. Finding Extreme Points in Contours with Open CV. https://pyimagesearch.com/2016/04/11/finding-extreme-points-in-contours-with-opencv/.

[34] Chollet F. (2017). Deep Learning with Python.

[35] Tan C., Sun F., Kong T., Zhang W., Yang C., Liu C. A survey on deep transfer learning. Proceedings of the International Conference on Artificial Neural Networks.

[36] Akram M.W., Li G., Jin Y., Chen X., Zhu C., Ahmad A. (2020). Automatic detection of photovoltaic module defects in infrared images with isolated and develop-model transfer deep learning. Sol. Energy.

[37] Ahmed W., Hanif A., Kallu K.D., Kouzani A.Z., Ali M.U., Zafar A. (2021). Photovoltaic Panels Classification Using Isolated and Transfer Learned Deep Neural Models Using Infrared Thermographic Images. Sensors.

[38] Oyetade I.S., Ayeni J.O., Ogunde A.O., Oguntunde B.O., Olowookere T.A. (2022). Hybridized deep convolutional neural network and fuzzy support vector machines for breast cancer detection. SN Comput. Sci..

[39] Budhiman A., Suyanto S., Arifianto A. Melanoma Cancer Classification Using ResNet with Data Augmentation. Proceedings of the 2019 International Seminar on Research of Information Technology and Intelligent Systems (ISRITI).

[40] Mahbod A., Schaefer G., Wang C., Dorffner G., Ecker R., Ellinger I. (2020). Transfer learning using a multi-scale and multi-network ensemble for skin lesion classification. Comput. Methods Programs Biomed..

[41] Ali K., Shaikh Z.A., Khan A.A., Laghari A.A. (2022). Multiclass skin cancer classification using EfficientNets—A first step towards preventing skin cancer. Neurosci. Inform..

[42] Carcagnì P., Leo M., Cuna A., Mazzeo P.L., Spagnolo P., Celeste G., Distante C. Classification of skin lesions by combining multilevel learnings in a DenseNet architecture. Proceedings of the Image Analysis and Processing—ICIAP 2019: 20th International Conference.

[43] Sevli O. (2021). A deep convolutional neural network-based pigmented skin lesion classification application and experts evaluation. Neural Comput. Appl..

[44] Zafar M., Amin J., Sharif M., Anjum M.A., Mallah G.A., Kadry S. (2023). DeepLabv3+-Based Segmentation and Best Features Selection Using Slime Mould Algorithm for Multi-Class Skin Lesion Classification. Mathematics.

[45] Bansal P., Garg R., Soni P. (2022). Detection of melanoma in dermoscopic images by integrating features extracted using handcrafted and deep learning models. Comput. Ind. Eng..

